# Evaluation of Intra-Articular Amikacin Administration in an Equine Non-inflammatory Joint Model to Identify Effective Bactericidal Concentrations While Minimizing Cytotoxicity

**DOI:** 10.3389/fvets.2021.676774

**Published:** 2021-05-21

**Authors:** Lynn Pezzanite, Lyndah Chow, Dean Hendrickson, Daniel L. Gustafson, A Russell Moore, Jason Stoneback, Gregg M. Griffenhagen, Gabriella Piquini, Jennifer Phillips, Paul Lunghofer, Steven Dow, Laurie R. Goodrich

**Affiliations:** ^1^Department of Clinical Sciences, College of Veterinary Medicine and Biomedical Sciences, Colorado State University, Fort Collins, CO, United States; ^2^Department of Microbiology, Immunology and Pathology, College of Veterinary Medicine and Biomedical Sciences, Colorado State University, Fort Collins, CO, United States; ^3^Department of Orthopedic Surgery, University of Colorado School of Medicine, Aurora, CO, United States

**Keywords:** intra-articular, antibiotic, aminoglycoside, amikacin, cartilage, horse

## Abstract

Septic arthritis causes significant morbidity and mortality in veterinary and human clinical practice and is increasingly complicated by multidrug-resistant infections. Intra-articular (IA) antibiotic administration achieves high local drug concentrations but is considered off-label usage, and appropriate doses have not been defined. Using an equine joint model, we investigated the effects of amikacin injected at three different doses (500, 125, and 31.25 mg) on the immune and cartilage responses in tibiotarsal joints. Synovial fluid (SF) was sampled at multiple time points over 24 h, the cell counts determined, and amikacin concentrations measured by liquid chromatography-mass spectrometry. Cytokine concentrations and collagen degradation products in SF were measured by ELISA and multiplex immunoassays. The mean amikacin concentrations in SF were greater than or equal to the minimum inhibitory concentration (MIC) (0.004 mg/ml) for most common equine joint pathogens at all time points tested to 24 h for all three amikacin doses evaluated. The inflammatory cytokines tumor necrosis factor-alpha (TNF-α) and interleukin-1 beta (IL-1β) increased significantly in SF in the highest amikacin dose group, despite the fact that increases in SF cell counts were not observed. Similarly, the biomarkers of cartilage type II collagen cleavage (C2C and C12C) were increased in SF following amikacin injection. Mechanistically, we further demonstrated using *in vitro* studies that chondrocytes and synoviocytes killed by exposure to amikacin underwent apoptotic cell death and were phagocytosed by macrophages in a non-inflammatory process resembling efferocytosis. Neutrophils and T cells were susceptible to amikacin cytotoxicity at clinically relevant doses, which may result in blunting of cellular inflammatory responses in SF and account for the lack of increase in total nucleated cell counts following amikacin injection. In summary, decisions on whether to inject cytotoxic antibiotics such as aminoglycosides intra-articularly and what doses to use should take into account the potential harm that antibiotics may cause and consider lower doses than those previously reported in equine practice.

## Introduction

Septic arthritis causes significant morbidity and mortality in both equine and human clinical practice (1, 2). The case fatality rates in humans are 11–15% in monoarticular disease, but increase to 50% when multiple joints are involved (1, 3–6). Reduced functional outcomes and osteomyelitis are reported in 24% and 8% of human survivors, respectively (7). The risk factors for developing septic arthritis in people include joint prosthesis, rheumatoid or osteoarthritis, skin disease, previous intra-articular corticosteroid injection, and comorbidities such as diabetes (1, 8–10). Regardless of age group or the risk factors present, *Staphylococcus aureus* is the most common causative agent in humans, followed by other Gram-positive bacteria (7, 9, 11). The incidence of septic arthritis in people is reportedly increasing, which has been attributed to a progressively aged population, higher number of invasive orthopedic procedures being undertaken, more frequent orthopedic-related infections, and increased use of immunosuppressive therapies (1, 8). Therefore, investigation of therapies to improve outcomes in septic arthritis is indicated.

Current treatments for septic arthritis in both human and equine patients include lavage with debridement of purulent material *via* arthroscopy or, less commonly, arthrotomy, antibiotics administered systemically and by regional perfusion, or, rarely, serial synovial fluid aspirations in patients too unstable to undergo general anesthesia (1, 2). However, unlike the case in human orthopedic practice, antibiotics have also been administered intra-articularly (IA) in equine veterinary medicine as an adjunctive therapy to treat septic arthritis for decades (12–16). This route of administration has more recently gained attention in human medical practice, particularly following prosthetic arthroplasty, in an attempt to address the issue of chronic biofilm infections with an increasing incidence of drug-resistant bacteria through achieving high local antibiotic concentrations (17–21). However, intra-articular antibiotic administration is “off-label” in all species, and appropriate intra-articular doses have not been defined, nor potential cytotoxicity with this route of administration investigated, for any of the major classes of antibiotics. Previous studies have demonstrated the potential for antibiotic cytotoxicity *in vitro* on joint cells from humans and veterinary species (17, 18, 22–24). Moreover, despite the widespread use of intra-articular antibiotics in equine practices, studies in horses have revealed variable toxicities following IA antibiotic administration depending on the antibiotic and the dose selected (25–32). However, no previous studies have compared antibiotic cytotoxicity or performed *in vivo* dose titration studies to assess the impact of antibiotics administered IA in any species on joint inflammation and cartilage degradation.

Therefore, we conducted a series of preliminary *in vitro* investigations to better define the capacity of different antibiotic classes to exert cytotoxic effects on chondrocytes and synovial cells from horses and dogs (22, 23). We demonstrated that amikacin, the antibiotic most commonly used intra-articularly in horses (12–14), was the most cytotoxic of the antibiotics evaluated to chondrocytes and rapidly induced apoptotic cell death (23). We built on that work in this study using a combination of *in vitro* assays and an *in vivo* equine model of intra-articular antibiotic administration to evaluate the potential impact of the amikacin doses used clinically in equine practice on overall joint health. We determined the pharmacokinetics of amikacin administered at multiple doses and quantified the biomarkers of inflammation and cartilage damage in the synovial fluid. To further assess macrophage responses to joint cells killed by amikacin, we generated equine monocyte-derived macrophage co-cultures and assessed the macrophage secretion of inflammatory cytokines following engulfment of amikacin-killed cells in a process known as efferocytosis. The overall goal of this work was to identify the amikacin doses that could achieve effective bacterial inhibitory concentrations for common joint pathogens while minimizing the induction of cytotoxicity and inflammation in the joint.

## Materials and Methods

### Horses

The use of six healthy 4- to 12-year-old Quarter Horses (four geldings, two mares) was approved by the Colorado State University (CSU) Institutional Animal Care and Use Committee (protocol no. 19-9058A). Horses were determined healthy by physical examination and lameness evaluation by board-certified veterinary surgeons (LG and LP) and radiographic examination (four-view) of both tarsi. All horses were sound in the hindlimbs at the trot and lacked radiographic evidence of osteoarthritis of the tarsal joints prior to study enrollment.

### Study Design

**Figure 1:**
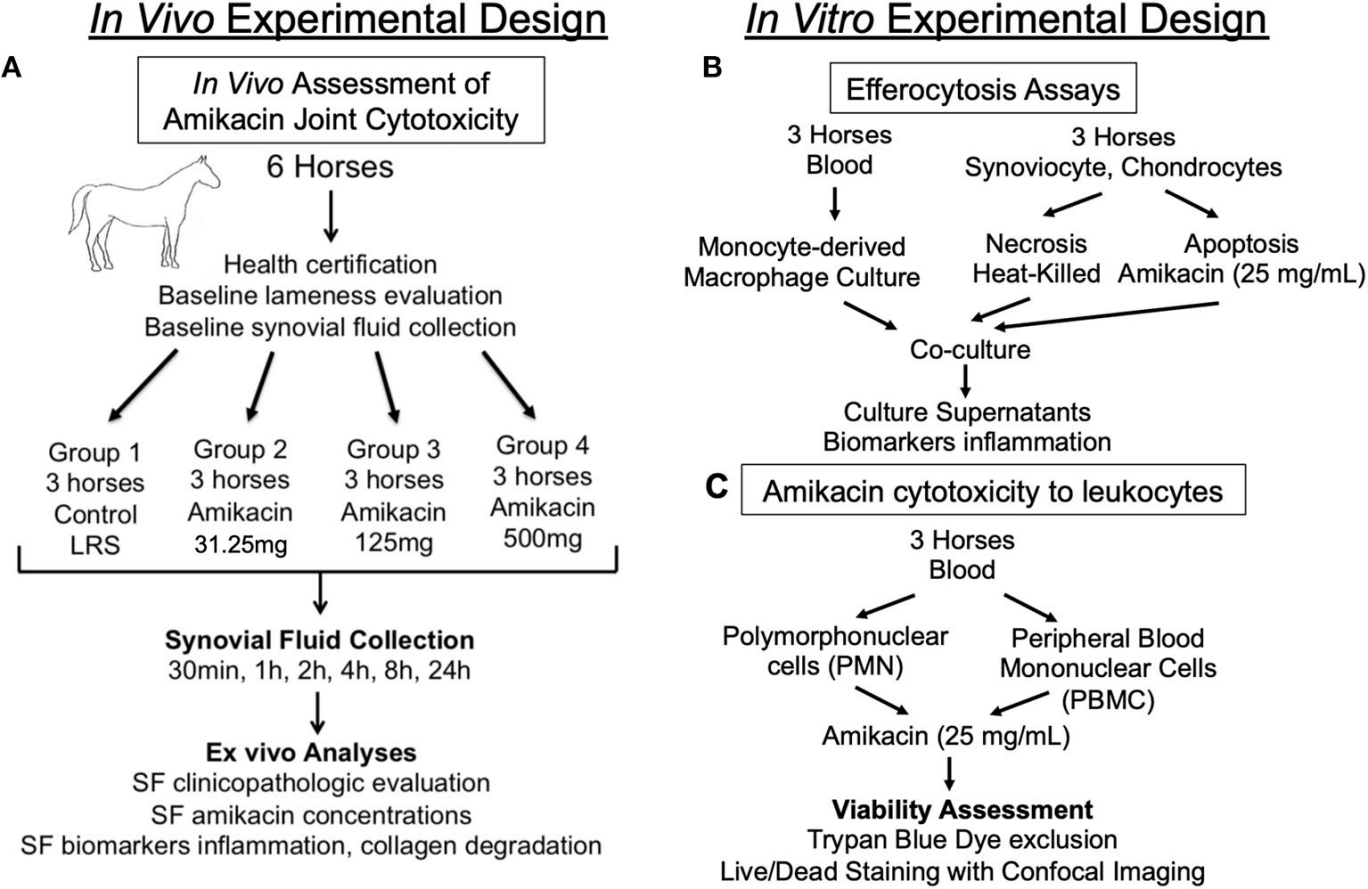
Diagram outlining the experimental design. Schematic overview of the study design for the **(A)** *in vivo* assessment of the cytotoxicity of amikacin on the joint of horses; **(B)** *in vitro* evaluation of macrophage efferocytosis of joint cells killed by amikacin; and **(C)** *in vitro* assessment of amikacin cytotoxicity to leukocytes. *SF*, synovial fluid.

This study was implemented with a randomized incomplete block design, with the random assignments being first leg treated (left or right), with two of the four treatments administered to each horse, and then the order that the treatments were administered. A random number generator (random.org) was used to randomize both the initially treated leg as well as which of the two treatments the horses received. Each horse received either amikacin at one of three doses (amikacin sulfate, 250 mg/ml; Teva Pharmaceuticals, Inc.) or lactated Ringer's solution (LRS) intra-articularly (Figure 1). The treatment groups were allocated as follows: group 1, 31.25 mg amikacin (diluted in LRS to a total volume of 2 ml); group 2, 125 mg amikacin (diluted in LRS to a total volume of 2 ml); group 3, 500 mg amikacin; and group 4, equivalent volume of LRS (2 ml) as the control group. Each horse was initially assigned to receive one treatment in either the left or the right tibiotarsal joint. After a 2-week washout period, the contralateral joint was injected with a second randomly assigned treatment. Randomization of treatment allocation and order was performed by one individual (LP) who was unblinded during the experiment; all other collaborators were blinded to the treatments throughout the data collection and analysis. Thus, six horses were administered two treatments each, but no two horses received the same pair of treatments, and each group included three horses in total (*n* = 3). The sample size of three horses per treatment allocation was determined based on previous literature (13).

Horses were sedated with a combination of xylazine (0.2–1 mg/kg, i.v.) or detomidine (0.01–0.02 mg/kg, i.v.) and butorphanol tartrate (0.01 mg/kg, i.v.) to effect. The tibiotarsal joint was aseptically prepared with chlorhexidine gluconate (4%, VetOne, MWI, Boise, ID, USA) followed by 70% ethanol and injected as described above. The highest dose was selected based on previous reports by equine practitioners of the use of up to 500 mg amikacin per joint, and lower titrated doses (125 and 31.25 mg) were also evaluated (24, 33).

### Synovial Fluid Sample Collection

Synovial fluid (SF) samples (1–2 ml) were aspirated from the injected tibiotarsal joint at baseline and at 0.5, 1, 2, 4, 8, and 24 h post-drug administration. Prior to each sample collection, the tibiotarsal joint was aseptically prepared and the horses were sedated as described above. SF was aspirated with a sterile 20-gauge needle into vials and either submitted immediately for clinicopathogical fluid analysis or aliquoted and stored at −80°C until later use in immunoassays or amikacin concentration determination *via* mass spectrometry for pharmacokinetic analysis.

### Synovial Fluid Clinicopathological Parameters

SF samples were submitted to the CSU Clinical Pathology Laboratory for evaluation by a board-certified clinical pathologist (ARM) for fluid analysis including total nucleated cell count (Hematrue, Heska Corp, Loveland CO, USA), refractometric total protein, preparation of Wright–Giemsa-stained (Aero-spray, Logan, UT, USA) direct smears for the determination of a manual leukocyte differential, and subjective grading of the glycosaminoglycans (adequate or disrupted) and quantity of red blood cells (within normal limits or excessive).

### Amikacin Concentration Determination

Extraction and analysis of amikacin from equine SF samples was performed using a high-pressure liquid chromatography/tandem mass spectrometry (LC/MS/MS) system. The system consisted of a 50-mm C_18_ column (2.1-mm i.d.) with a Phenomenex C18 Filter Frit Guard Cartridge on the Shimadzu HPLC system coupled to the 3200 A-TRAP triple quadrupole mass spectrometer (Applied Biosystems, Inc., Foster City, CA, USA) with a flow rate of 750 μl/min. The instrument was operated in multiple reaction monitoring (MRM) positive ion mode. The LC gradient conditions (ion-pairing chromatography) were 10 mM heptafluorobutyric acid with 10 mM ammonium hydroxide in Milli-Q water (mobile phase A) and 5 mM heptafluorobutyric acid with 5 mM ammonium hydroxide in 10:90 Milli-Q water/acetonitrile (mobile phase B).

Amikacin stock solution (100 mg/ml) in 10 mM heptafluorobutyric acid with 10 mM ammonium hydroxide diluted in Milli-Q water was prepared. A standard curve (10, 25, 50, 75, 100, 250, 500, 750, 1,000, 2,500, 5,000, and 10,000) of amikacin (in micrograms per milliliter) in Milli-Q water was prepared. A 1-mg/ml solution of amikacin in 10 mM ammonium hydroxide in Milli-Q water was prepared, and 40 ml of the final dilution solution (FDS) (50/50) mobile phase B/Milli-Q with 2,500 ng/ml amikacin was used as an internal standard. Blank equine synovial fluid (45 μl) was added to 1.5 ml microcentrifuge tubes as dilution standards, vortexed for 1 min, and centrifuged for 5 min at 14,000 rpm. Standard dilutions (1:100, 1:1,000, and 1:10,000) in Milli-Q water were prepared by transferring 10 μl of each standard to 990 μl of Milli-Q water in 1.5 ml microcentrifuge tubes. Six QC samples (3 × 25, 3 × 100, and 3 × 750 μg/ml) were prepared, vortexed for 5 min, and centrifuged for 1 min at 8,000 × *g*. Standard samples (125 μl) were transferred into sample vials and analyzed.

To analyze the amikacin concentrations in SF samples, dilutions (1:100, 1:1,000, and 1:10,000) were prepared using Milli-Q in 1.5 ml microcentrifuge tubes. The samples were vortex mixed for 5 min, centrifuged for 1 min at 8,000 × *g*, and transferred into autosample vials for analysis.

### Determination of Biomarkers of Cartilage Metabolism

Competitive ELISAs, previously validated for equine samples (IBEX Pharma, Quebec, Canada), were used to measure the concentrations of biomarkers C2C (biomarker of type II collagen degradation) and C12C [biomarker of type I (soft tissue) and type II (cartilage) collagen degradation] in the SF from all treatment groups collected at time points 0, 8, and 24 h as previously described (34).

### Determination of Biomarkers of Joint Inflammation (Cytokines and Collectin)

The concentrations of C-reactive protein (CRP), a widely accepted inflammatory marker, were measured using competitive ELISA, previously validated for use in equine samples (Immunology Consultant Laboratories, Portland, OR, USA), from all treatment groups at time points 0, 8, and 24 h. A fluorescent bead-based multiplex assay (MILLIPLEX MAP Equine Cytokine/Chemokine Magnetic Beads Multiplex Assay, Millipore Sigma, Burlington, MA, USA) was used to quantify the concentrations of 23 analytes [eotaxin/CCL11, fibroblast growth factor 2 (FGF-2), fractalkine/CS3CL1, granulocyte colony-stimulating factor (G-CSF), granulocyte–macrophage colony-stimulating factor (GM-CSF), GRO, interferon (IFN), interleukin (IL)-1α, IL-1β, IL-2, IL-4, IL-5, IL-6, IL-8/CXCL8, IL-10, IL-12, IL-13, IL-17a, IL-18, IP-10, MCP-1, RANTES/CCL5, and tumor necrosis factor alpha (TNF-α)] in the SF from all time points.

### *In vitro* Culture of Equine Macrophages

To generate macrophages, equine peripheral blood mononuclear cells were isolated from whole blood of three horses (Quarter Horses, aged 2–3 years, one mare and two geldings) *via* density gradient centrifugation (Ficoll-Paques™ PLUS, GE Healthcare Bio-Sciences) and cultured in macrophage medium [Dulbecco's modified Eagle's medium (DMEM) supplemented with 10% fetal bovine serum, non-essential amino acids, and penicillin/streptomycin antibiotics; Sigma-Aldrich] with human M-CSF (PeproTech, Rocky Hill, NJ, USA) at 25 ng/ml to stimulate differentiation into macrophages in 3–5 days, as previously described (35).

### Efferocytosis and Cytokine Suppression Assays

Equine synoviocytes and chondrocytes were collected postmortem and isolated from three different horses euthanized for reasons unrelated to the study (Quarter Horses, aged 2–3 years, two geldings and one mare) and expanded in culture as previously described (22). The synoviocytes and chondrocytes were killed rapidly at passage 1 by exposure to either amikacin (25 mg/ml, diluted in DMEM supplemented with 10% fetal bovine serum and penicillin/streptomycin antibiotics, representing apoptotic cell death) or heat [heat killed (HK), 50°C water bath, representing necrotic cell death] and then co-cultured at a 1:1 ratio with equine monocyte-derived macrophages in monolayer culture for 2 h. After 2 h in culture with dead cells to allow time for phagocytosis, the macrophages were washed in phosphate-buffered saline and allowed to culture in growth media for an additional 18 h. The supernatants were collected at that time for the evaluation of cytokines on a limited panel multiplex bead assay (IL1-β, IL-6, TNF-α; MilliporeSigma, Burlington, MA, USA) and transforming growth factor beta 1 (TGF-β1) ELISA (R&D Systems, Minneapolis, MN, USA).

### Amikacin Cytotoxicity on Polymorphonuclear Cells and Peripheral Blood Mononuclear Cells

Three different horses (Quarter Horses, aged 2–3 years, one gelding and two mares) donated blood for the isolation of peripheral blood mononuclear cells (PBMCs) and polymorphonuclear cells (PMNCs), and the cytotoxicity of amikacin on each cell line was assessed. PBMCs were isolated *via* density gradient centrifugation (Ficoll-Paque™ PLUS, GE Healthcare Bio-Sciences) at 400 × *g* for 30 min. PMNCs were isolated using the Lympholyte-Poly (Cedarlane, Peterborough, UK) separation gradient according to the manufacturer's instructions. The PBMCs and PMNs from the three horses were exposed to amikacin over a range of concentrations in complete growth medium (25, 12.5, 6.25, 3.125, 1.56, 0.78, and 0.39 mg/ml vs. control) in triplicate for 24 or 1 h, respectively. Live/dead visualization of PBMCs following amikacin exposure was further performed using the LIVE/DEAD Viability and Counting Kit (Thermo Fisher Scientific) according to the manufacturer's instructions and visualized on an Olympus IC83 confocal microscope. Ratios of live to dead cells were calculated by imaging the total area of each channel using ImageJ software (36).

### Data Analysis and Pharmacokinetic Modeling

Statistical comparisons between the four treatment groups used ANOVA, followed by Tukey's adjustment for multiple comparisons. Data and residuals were visually assessed for normality. All data points collected were included in the final analysis. Significant differences between the clinicopathological parameters and biomarkers were evaluated between baseline and at each time point using a two-way ANOVA with repeated measures. The appearance of glycosaminoglycans, granulated small monocytes, or excessive red blood cells over the range of amikacin concentrations and time was analyzed using logistic regression (function “*glm*” from the base STATS package). Biomarkers were normalized to baseline for analysis due to the variability between the baseline values between individual horses.

The half-maximal inhibitory concentration (IC_50_), or the concentration of antibiotic at which 50% of cells (PBMCs and PMNCs) were viable, was determined by normalizing the dose response for each concentration to the control, transforming the data to normalized dose response vs. log10 (concentration) and estimating using IC_50_ non-linear regression by fitting the data to a three-parameter sigmoid function [implemented as “log(inhibitor) vs. dose response”]. In instances where the IC_50_ was calculated to be outside the range of the concentrations evaluated, or the data were not distributed in a sigmoid fashion following log transformation, the IC_50_ data were reported as a range of values as the exact value could not be determined based on the concentrations assessed.

The pharmacokinetic parameters for each individual dose level were calculated by non-compartmental analysis using the PKNCA package for R (37). The ANOVA and IC_50_ calculations were performed using Prism software v8.4.1 (GraphPad Software Inc., La Jolla, CA, USA). Logistic regression and pharmacokinetic analyses were performed using R v4.0.0 (“Arbor Day,” R Foundation for Statistical Computing, Vienna, Austria). Significance was set at *p* ≤ 0.05.

## Results

### Defining Amikacin Pharmacokinetics Following Intra-Articular Administration

**Figure 2:**
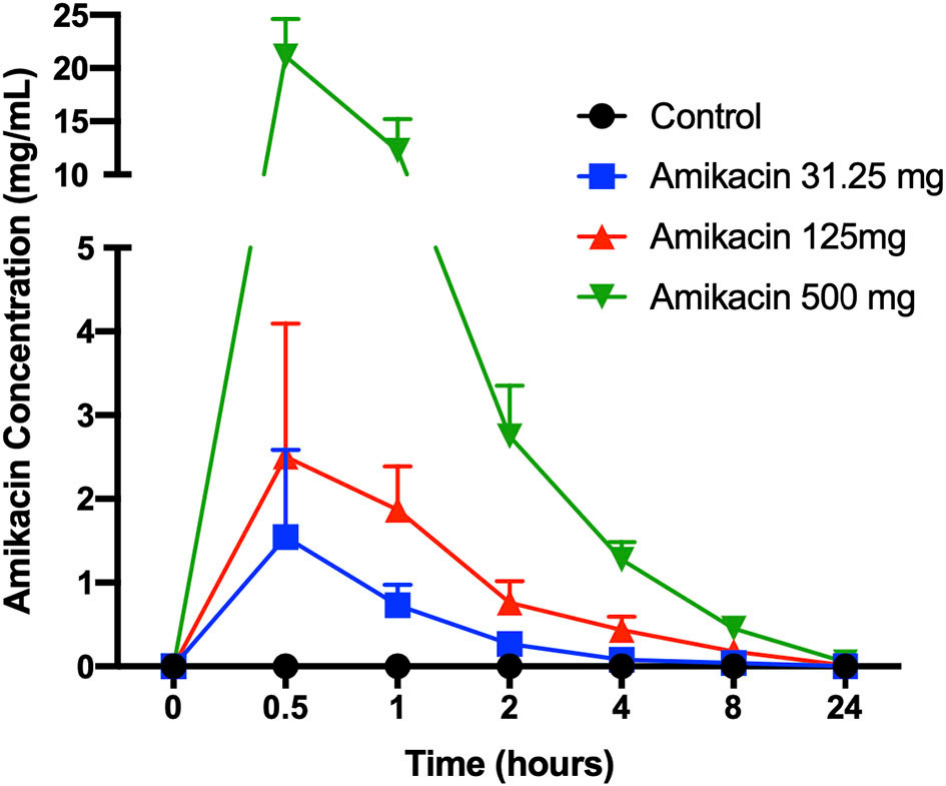
Synovial fluid amikacin concentrations over time following a single intra-articular administration of amikacin at three concentrations. Synovial fluid amikacin concentrations (*n* = 3 horses per group) were determined by high-pressure liquid chromatography/mass spectrometry and expressed as the mean ± SD.

Synovial fluid amikacin concentrations were detectable for all doses injected at all time points evaluated, and the mean remained greater than or equal to the minimum inhibitory concentration (MIC) for most common equine pathogens (>4 μg/ml) at 24 h (Figure 2). The pharmacokinetic data (*c*_max_, half-life, AUC_last_, and AUC_inf_pred_) are reported in Table 1. The amikacin values at each time point (mean ± SD) are reported in Table 2.

**Table 1:** Mean ± SD synovial fluid amikacin concentrations over 24 h following a single intra-articular administration of amikacin at one of three doses (500, 125, or 31.25 mg) or the control (LRS, *n* = 3 horses per group).

	**Treatment**							
	**Control**		**Amikacin 31.25 mg**		**Amikacin 125 mg**		**Amikacin 500 mg**	
	**Mean (mg/ml)**	**SD**	**Mean (mg/ml)**	**SD**	**Mean (mg/ml)**	**SD**	**Mean (mg/ml)**	**SD**
**Time (h)**								
0	0.000	0.000	0	0	0.000	0.000	0.000	0.000
0.5	0.000	0.000	1.545	1.041	2.503	1.590	21.100	3.500
1	0.000	0.000	0.732	0.244	1.870	0.517	12.233	2.967
2	0.000	0.000	0.266	0.113	0.761	0.257	2.750	0.601
4	0.000	0.000	0.078	0.010	0.435	0.157	1.273	0.211
8	0.000	0.000	0.040	0.012	0.177	0.120	0.450	0.141
24	0.000	0.000	0.004	0.001	0.012	0.005	0.047	0.032

**Table 2:** Pharmacokinetic parameters evaluated in the synovial fluid following a single intra-articular administration of amikacin at one of three doses to six Quarter Horses.

**Amikacin dose (mg)**	***c***_**max**_ **(ug/ml)**	**Half-life (h)**	**AUC**_**last**_ **(ug/ml)**	**AUC**_**inf_pred**_ **(ug/ml)**
31.25	1,680	4.58	2,210	2,230
125	3,230	3.79	6,120	6,180
500	20,900	4.33	29,300	29,600

*All values were generated using non-compartmental analysis. c_max_, maximum measured concentration; AUC_last_, area under the synovial fluid concentration curve from time 0 to the last measurable concentration; AUC_0−inf_, area under the synovial fluid concentration curve extrapolated to infinity*.

### Impact of Intra-Articular Amikacin on Biomarkers of Cartilage Metabolism and Health

**Figure 3:**
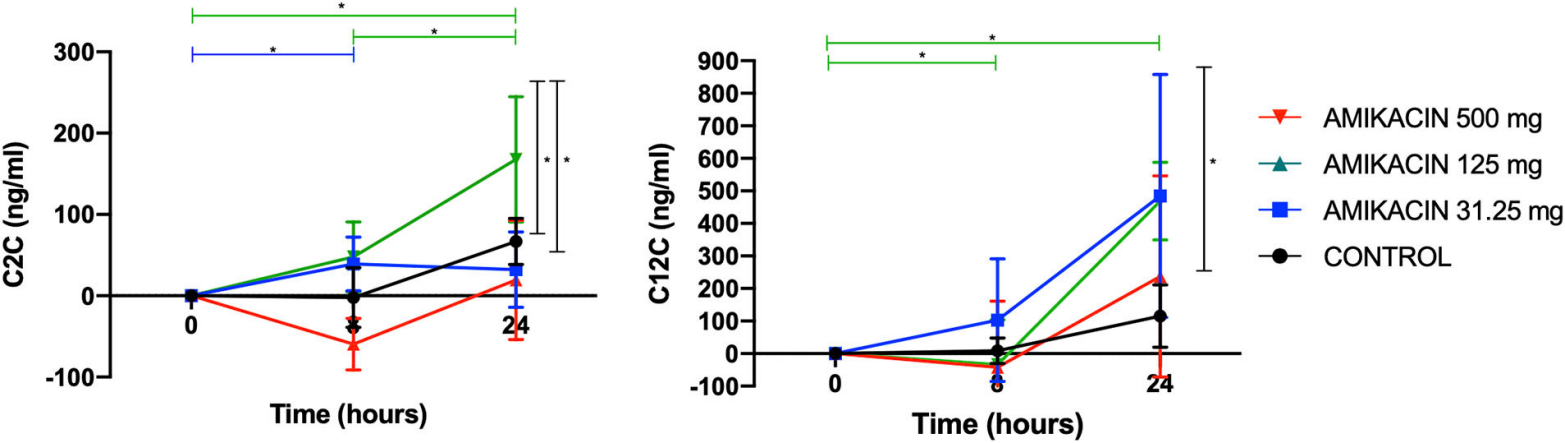
Concentrations of the biomarkers of collagen degradation following a single intra-articular administration of amikacin at three doses compared to the control, normalized to baseline. Competitive enzyme-linked immunosorbent assays (ELISAs), previously validated for use in equine synovial fluid, were used to measure the biomarker concentrations of neo-epitope C2C (biomarker of type II collagen degradation) and C12C [biomarker of type I (soft tissue) and type II (cartilage) collagen degradation]. Results are expressed as the mean ± SD, normalized to baseline.

Immunoassay revealed dose-dependent increases in the C2C levels, which were increased in horses treated with 125 mg amikacin at 8 h vs. baseline (*p* = 0.02), horses receiving 500 mg amikacin at 24 h vs. baseline (*p* = 0.01), and at 24 vs. 8 h (*p* = 0.05). When evaluated at each time point, the C2C levels had a high magnitude of change between the treatment groups at 8 h for 31.25 vs. 125 mg (*p* = 0.002) and for 125 vs. 500 mg (*p* = 0.005) and at 24 h for 31.25 vs. 500 mg (*p* = 0.03) and for 125 vs. 500 mg (*p* = 0.04) (Figure 3).

The C12C levels increased in horses treated with 500 mg amikacin at 24 h vs. baseline (*p* = 0.0006) and at 24 vs. 8 h (*p* = 0.006). When evaluated at each time point, C12C was increased with 500 mg amikacin *vs*. control at 24 h (*p* = 0.002) (Figure 3).

Glycosaminoglycan content was not different between groups (*p* = 0.95), but was disrupted with increasing frequency over time with repeated arthrocentesis in all treatment groups (*p* < 0.001).

### Impact of Intra-Articular Amikacin Administration on Cytokine and Collectin Concentrations in SF

**Figure 4:**
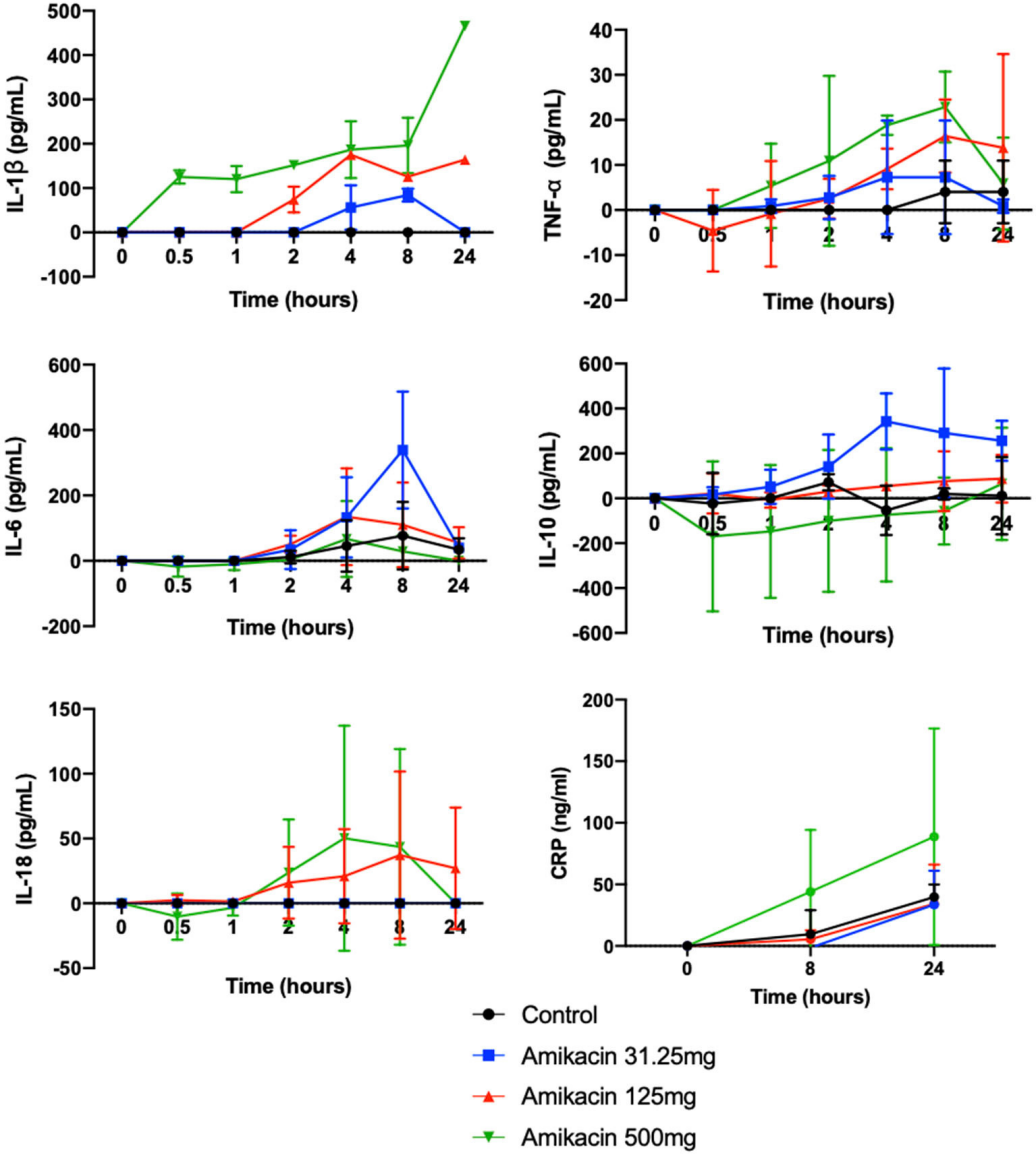
Concentrations of the biomarkers of inflammation in synovial fluid of joints injected with amikacin, normalized to baseline. The concentrations of 23 analytes (eotaxin/CCL11, FGF-2, fractalkine/CS3CL1, G-CSF, GM-CSF, GRO, IFN, IL-1α, IL-1β, IL-2, IL-4, IL-5, IL-6, IL-8/CXCL8, IL-10, IL-12 (p70), IL-13, IL-17a, IL-18, IP-10, MCP-1, RANTES/CCL5, and TNFα) were quantified in synovial fluid samples from all treatment groups at all time points using fluorescent bead-based multiplex assay. Measurable cytokine levels were detectable for 10 of 23 cytokines (IL1β, FGF, G-CSF, IL-10, TNFα, IL-6, IL-8, IL-18, IP-10, or MCP-1). The cytokine levels were compared between treatment groups at each time point and between time points for each treatment group, as well as for the overall effect of treatment and time by two-way analysis of variance. Significant differences were seen across time points for IL1-β for control vs. 125 mg amikacin (*p* < 0.0008) and control vs. 500 mg (*p* < 0.0001), TNF-α for control vs. 500 mg (*p* = 0.0281), and IL-10 for control vs. 31.25 mg amikacin (*p* = 0.0066). Five cytokines (IL1β, FGF, G-CSF, IL-10, and TNFα) additionally had significantly different levels between time points for each treatment group evaluated or between treatment groups at different time points. No significant differences between treatment groups were observed in the levels of IL-6, IL-8, IL-18, IP-10, or MCP-1. An ELISA was also used to determine the concentrations of inflammatory C-reactive protein (CRP) in the synovial fluid. Three joints were included per treatment group. The cytokine levels were compared between three treatment groups using a two-way ANOVA. Results are expressed as the mean ± SD, normalized to baseline.

Cytokine multiplex analysis was used to assess the impact of amikacin on the SF cytokine concentrations over time (Figure 4 and Table 3). CRP concentrations increased in horses treated with 500 mg amikacin compared to all other treatments at 8 and 24 h, which did not reach statistical significance (concentration effect overall, *p* = 0.08) (Figure 3). Multiplex assay documented detectable levels for 10 of 23 cytokines (IL-1β, FGF, G-CSF, IL-10, TNF-α, IL-6, IL-8, IL-18, IP-10, or MCP-1). Five cytokines (IL-1β, FGF, G-CSF, IL-10, and TNF-α) had different levels between time points for each treatment group evaluated or between treatment groups at different time points (Figure 4 and Table 3). IL-1β was higher across time points when 125 mg was injected *vs*. control (*p* = 0.0008) and 500 mg vs. control (*p* < 0.0001). TNF-α levels were higher in samples with 500 mg amikacin *vs*. control (*p* = 0.03). The levels of IL-10 were higher in 31.25 mg amikacin *vs*. control (*p* = 0.007). No differences between treatment groups were observed in the levels of IL-6, IL-8, IL-18, IP-10, or MCP-1.

**Table 3:** Statistical analysis of the cytokine levels in the synovial fluid following amikacin treatment at various doses or the control (lactated Ringer's solution).

**Treatment**	**IL1-β**		**TNF-α**		**IL-6**		**IL-10**		**IL-18**	
Control vs. amikacin 31.25 mg	ns	0.10	ns	0.78	ns	0.35	^*^	0.007	ns	0.18
Control vs. amikacin 125 mg	^*^	0.0008	ns	0.43	ns	0.62	ns	0.53	ns	0.46
Control vs. amikacin 500 mg	^*^	<0.0001	^*^	0.03	ns	0.81	ns	0.54	ns	0.18

*Cytokine levels (IL-1β, TNF-α, IL-6, IL-10, and IL-18) were compared across time points between treatment groups and the control by two-way ANOVA with repeated measures*.

Significance was assessed at ^*^p ≤ 0.05. ns indicates non-significance statistically (significance assessed at p ≤ 0.05). Values indicated are the p-values.

### Synovial Fluid Clinicopathological Parameters

**Figure 5:**
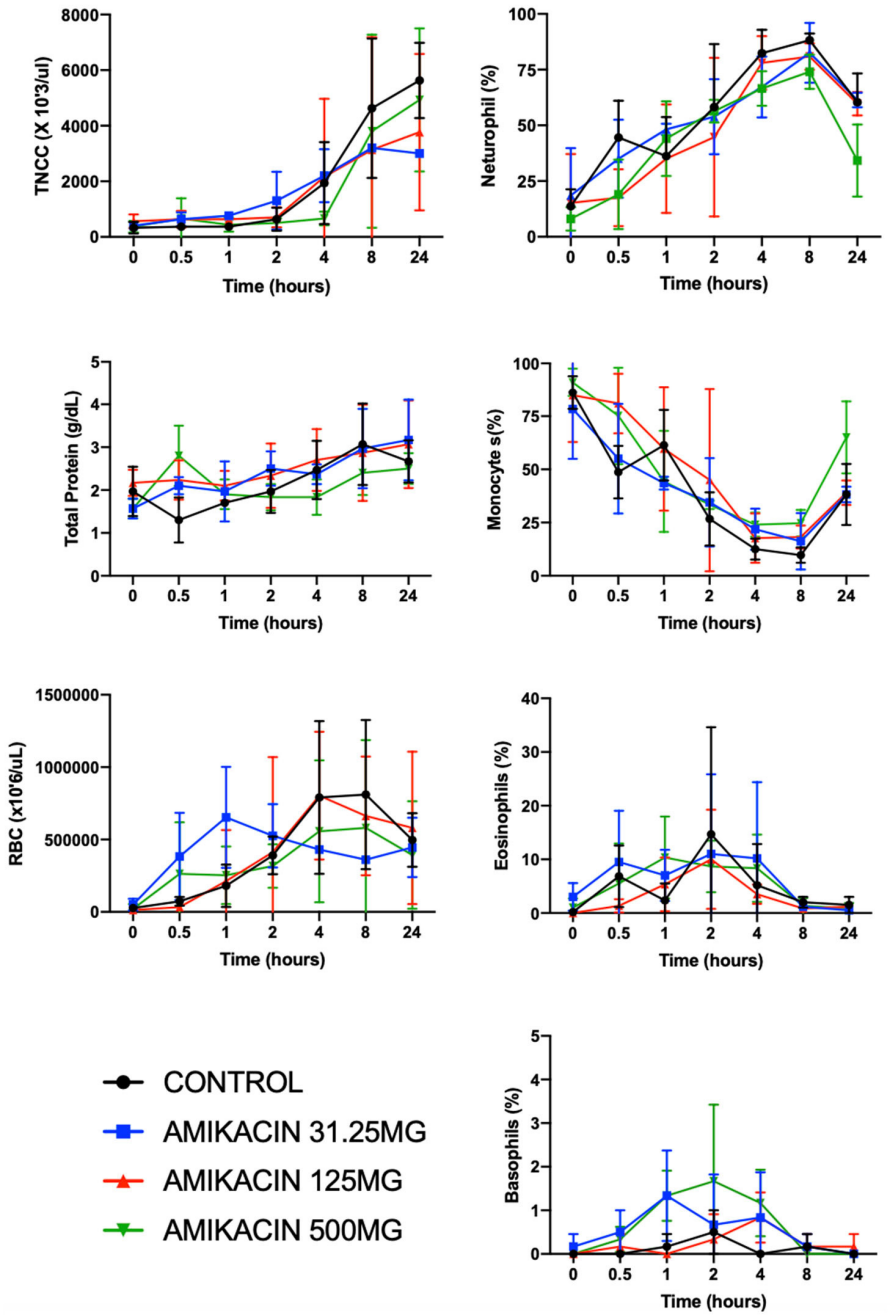
Clinicopathologic parameters for synovial fluid following a single intra-articular administration of amikacin in three treatment groups. Fluid analysis on synovial fluid samples obtained from three horses per group was performed on all samples for total nucleated cell count, total protein, and red blood cell count and leukocyte differential (*y*-axes are not equivalent across graphs). There were significantly increased neutrophils (in percent) and decreased monocytes (in percent) with repeated arthrocenteses over time. Results are expressed as the mean ± SD.

No differences were found between the treatment groups and controls with respect to total nucleated cell counts, total protein, and red blood cell count at each time point evaluated (*p* = 0.35, *p* = 0.70, and *p* = 0.14, respectively) (Figure 5). However, differences were seen between time points with repeated synoviocenteses (total nucleated cell count, *p* = 0.0002; total protein, *p* = 0.001; red blood cell count, *p* = 0.02). There were no differences between the amikacin treatments for each of the four cell types (neutrophils, *p* = 0.14; monocytes, *p* = 0.50; eosinophils, *p* = 0.89; basophils, *p* = 0.14), but there were differences for time with repeated synoviocenteses for each cell line (neutrophils, *p* < 0.0001; monocytes, *p* < 0.0001; eosinophils, *p* = 0.02; basophils, *p* = 0.03) (Figure 5). The presence of granulated small monocytes and excessive red blood cells was not different between treatment groups (*p* = 0.41 and *p* = 0.05, respectively). The presence of granulated small monocytes did not differ over time (*p* = 0.74). The presence of excessive red blood cells increased over time (*p* = 0.002).

### Mechanisms of Amikacin Cytotoxicity and Impact on Cell Clearance by Macrophages

**Figure 6:**
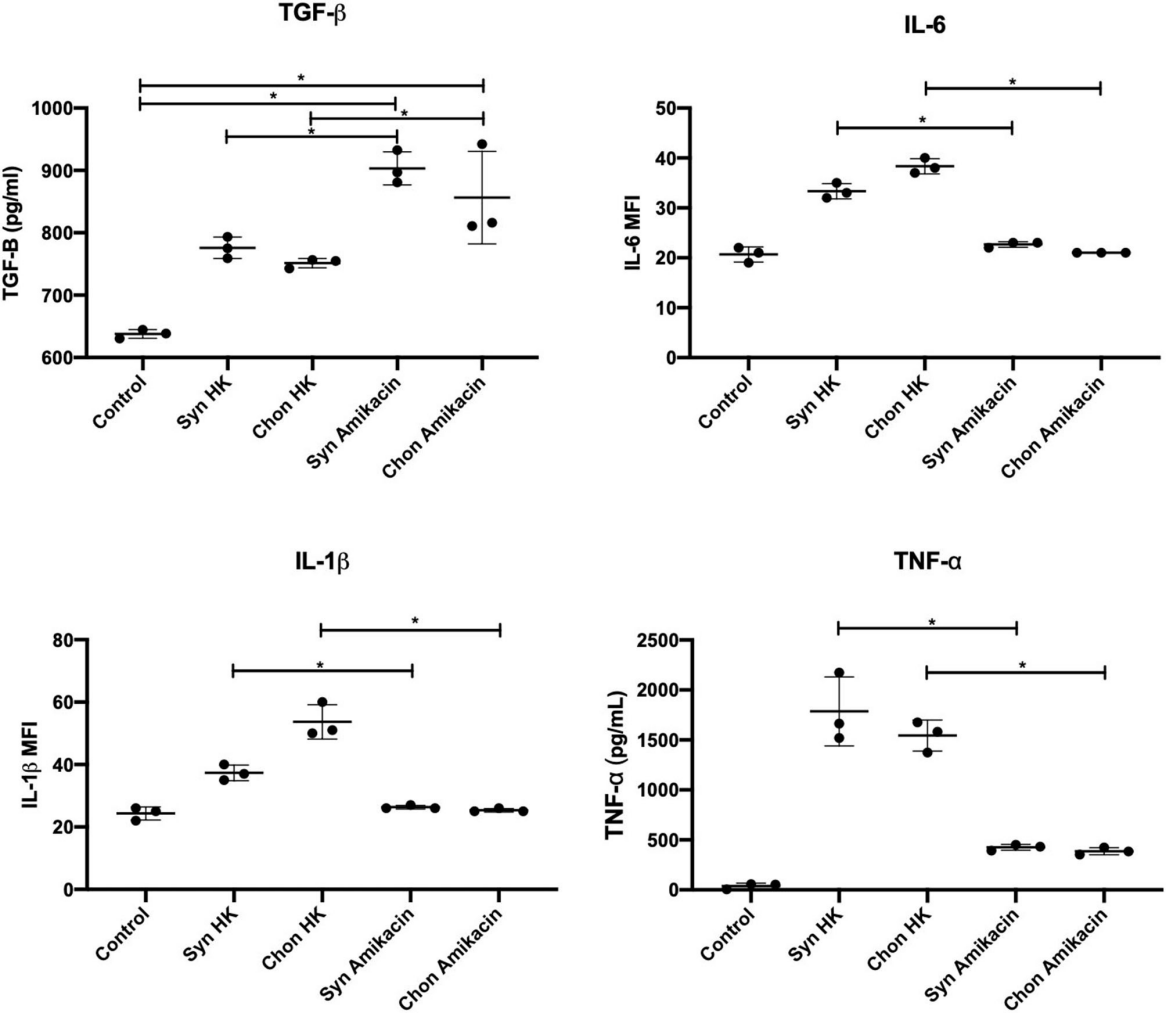
Efferocytosis and cytokine suppression assays quantifying IL1-β, IL-6, TNF-α, and TGF-β1 in macrophage culture media after incubation with equine synoviocytes and chondrocytes killed by induced apoptosis (25 mg/ml amikacin) or necrosis (heat killed, *HK*). Data were evaluated by two-way ANOVA. Significance was assessed at ^*^p ≤ 0.05.

Macrophages engulfing amikacin-killed cells demonstrated a reduced release of pro-inflammatory cytokines (IL-1β, TNF-α, and IL-6) and a concomitant increased production of anti-inflammatory cytokines (TGF-β) (Figure 6). For example, secretion of TGF-β1 was increased following efferocytosis of amikacin-killed synovial lining cells compared to necrotic cells (*p* = 0.01) Similarly, amikacin-killed chondrocytes also induced more TGF-β than did heat-killed chondrocytes (*p* = 0.03). In contrast, we observed that less IL-1β was produced by macrophages incubated with amikacin-killed cells than by macrophages incubated with heat-killed cells (*p* = 0.006). This was interpreted as evidence of efferocytosis of apoptotic cells, as previously described (38). Similar responses were noted for TNF-α (*p* < 0.0001) and IL-6 (*p* < 0.0001) production between amikacin- vs. heat-killed cells. These findings indicate that cell death induced by amikacin is inherently anti-inflammatory compared to the pathways associated with necrosis. These findings *in vitro* help explain why SF from amikacin-treated horses manifested a relatively benign response to ongoing cellular death and tissue injury locally within the joint.

### Relative Susceptibility of Leukocytes and Joint Cells to Amikacin Cytotoxicity

**Figure 7:**
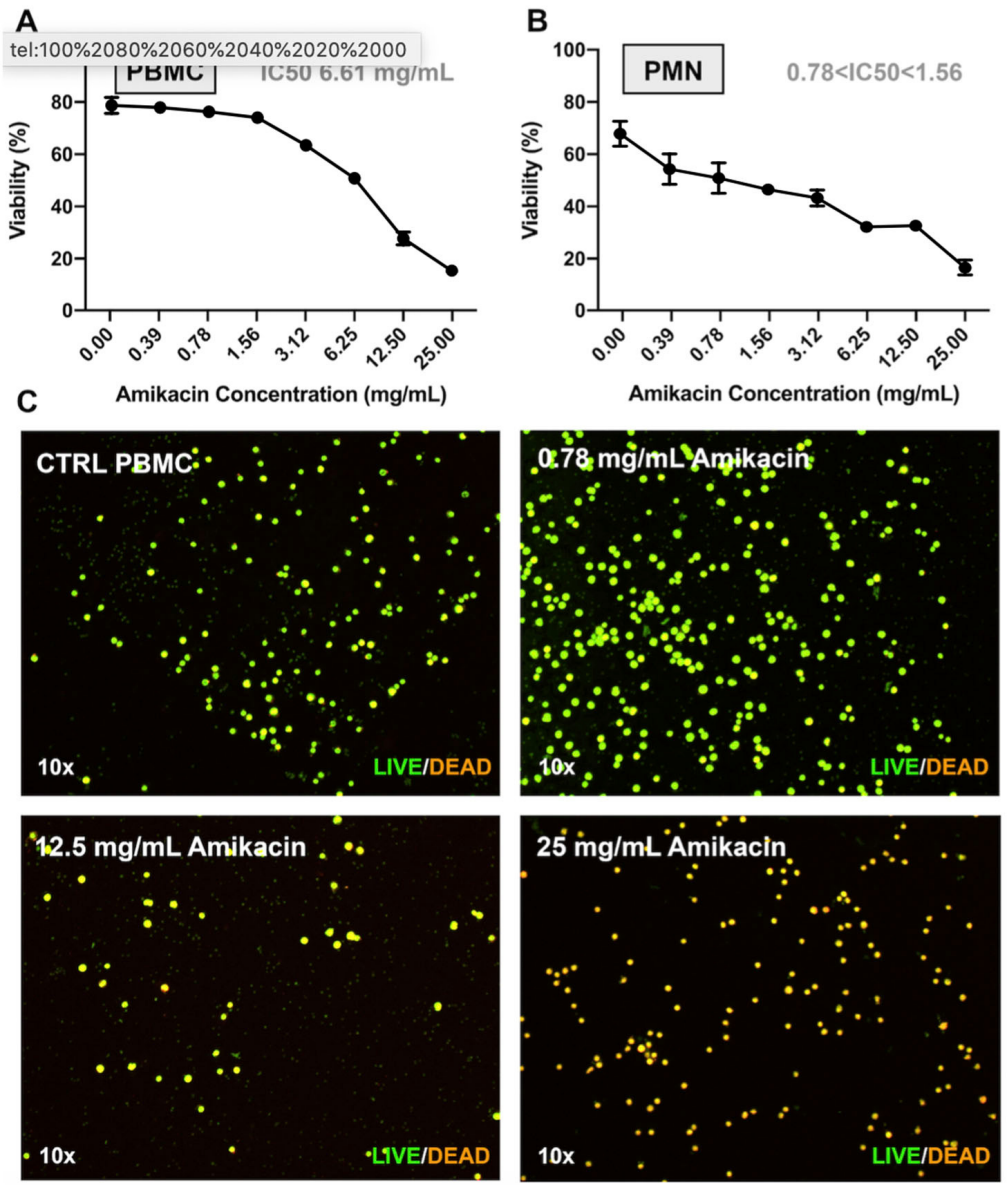
Effect of amikacin on neutrophils and peripheral blood mononuclear cells at clinically relevant doses. Cytotoxicity of amikacin on **(A)** peripheral blood mononuclear cells (PBMCs) and **(B)** polymorphonuclear cells (PMNs) derived from whole blood of three horse donors each in triplicate and assessed by Trypan blue dye exclusion over a range of amikacin concentrations. The inhibitory concentration 50 (IC_50_), or the concentration at which 50% of the cells were dead, is denoted. **(C)** Live/dead visualization of PBMCs following amikacin exposure over a range of doses reached clinically (0.78, 12.5, and 25 mg/ml) performed using the LIVE/DEAD Viability and Counting Kit (Thermo Fisher Scientific).

The preceding findings suggested another potential mechanism to dampen joint responses to amikacin toxicity. The SF white blood cell counts were not increased in amikacin-treated horses relative to the control, despite evidence of inflammation and cartilage damage (Figures 3–5). This could be explained, for example, if leukocytes, especially neutrophils, were particularly susceptible to amikacin toxicity. Therefore, we determined the mean cytotoxicity-inducing concentrations for purified populations of equine neutrophils and lymphocytes. The IC_50_ was 6.62 mg/ml for lymphocytes and between 0.78 and 1.56 mg/ml for neutrophils, demonstrating that lymphocyte toxicity would be induced at the concentrations achieved clinically following amikacin injection. Live/dead staining of PBMC following amikacin exposure demonstrated a dose-dependent cytotoxicity of amikacin-exposed PBMC at 4 h vs. the control (Figure 7). Thus, these findings suggest that the absence of a significant rise in SF leukocytes following amikacin injection may be attributed in part to the direct and rapid leukocyte killing *in situ* by high concentrations of amikacin, followed by rapid clearance by synovial macrophages.

## Discussion

Intra-articular antibiotics have been used for decades in equine practice and have been more recently reported in humans to treat orthopedic device-related infections following arthroplasty (19–21). As all intra-articular antibiotic use is “off-label,” appropriate doses have not been determined and potential cytotoxicity by this route of administration has not been fully assessed. Amikacin sulfate is the antibiotic most frequently used in joints in equine clinical practice, and previous work has further demonstrated it to be cytotoxic to equine cartilage *in vitro* (22, 23). For these reasons, we evaluated the cytotoxicity of three doses of amikacin administered in the tarsocrural joint of horses toward the goal of providing dosing guidelines for practitioners, with translational relevance to physicians who may also inject antibiotics in joints. Intra-articular administration of amikacin at doses currently used in horses induced both chondrotoxicity and inflammation in this model. Pharmacokinetic analysis of the synovial fluid indicated that lower amikacin doses than those frequently used in equine practice could achieve effective MICs while minimizing potential toxicity. We further investigated the mechanisms by which amikacin-induced inflammation was resolved within the joint using *in vitro* co-cultures of equine monocyte-derived macrophages with chondrocytes and synoviocytes and demonstrated it to be through a process resembling efferocytosis, further suppressing inflammation.

Amikacin sulfate is injected prophylactically in equine joint injections and to treat septic arthritis prior to receipt of bacterial culture and sensitivity due to its broad spectrum of activity against both Gram-positive and Gram-negative bacterial isolates, including *Staphylococcus, Escherichia, Enterobacter*, and *Pseudomonas* (39). This study was the first to evaluate an *in vivo* dose titration for intra-articular amikacin administration in horses, building on previous veterinary literature regarding amikacin pharmacokinetics. Sedrish et al. reported that SF amikacin concentrations remained greater than or equal to the MIC (4 μg/ml) for 24 h when 500 mg was injected into a normal equine radiocarpal joint (12). Taintor et al. further demonstrated that joint inflammation accelerated amikacin distribution from the joint, as amikacin (500 mg, radiocarpal joint) remained greater than or equal to the MIC in normal joints for 72 h, but only 48 h in endotoxin-inflamed joints, which was attributed to the differential antibiotic movement from the joint with increased synovial vascularity in inflammation (14). In this study, the mean amikacin concentrations in the synovial fluid reached levels well over 100× the MIC, and the mean levels were sustained at or above the established MIC for most common equine pathogens (4 μg/ml) for at least 24 h following injection of all the amikacin doses assessed (31.25, 125, and 500 mg) (40). While it is recognized that select equine organisms have been reported to have higher MICs (e.g., coagulase-positive *Staphylococcus* sp., 8 μg/ml; *Pseudomonas aeruginosa*, 64 μg/ml; and *Streptococcus zooepidemicus*, 128 μg/ml), these findings would suggest that amikacin doses lower than those commonly used by equine practitioners (125–500 mg) may be utilized to both reach and sustain therapeutic levels, although further evaluation in inflamed or infected joints is warranted (14, 33).

The degree of inflammation induced by amikacin injection and the subsequent mechanisms of resolution of inflammation warrant further discussion. Clinicopathological analysis of synovial fluid samples revealed alterations in differential cellular compositions over time with repeated synoviocenteses, but no differences between treatment groups. This may be attributed to the frequency of SF sampling, resulting in elevated total nucleated cell counts and total protein as well as an altered leukocyte differential, which could have overwhelmed any individual treatment effect. However, amikacin injection elicited dose-dependent increases in the pro-inflammatory cytokines TNF-α and IL1-β in the joint, indicating an inflammatory response. The concentrations of IL-10 were also elevated in the synovial fluid of horses injected with the lowest dose of amikacin; as IL-10 secretion is typically suppressed by IL-1β, this may be attributed to the less significant IL-1β downregulation of IL-10 at lower amikacin concentrations.

We explored this further to determine the mechanism by which amikacin-induced inflammation was resolved using *in vitro* assays to demonstrate phagocytosis and the clearance of joint cells killed by amikacin *via* equine macrophages. Dead and dying cells are rapidly cleared in the body by tissue macrophages through a process known as efferocytosis, and the mechanism of cell death (e.g., apoptosis and necrosis) significantly impacts how macrophages respond to these phagocytosed cells (38). In general, macrophage phagocytosis of apoptotic cells leads to the suppression of inflammation, while engulfment of necrotic cells provides a strong inflammatory stimulus (41). Therefore, we conducted *in vitro* experiments to elucidate the mechanisms by which amikacin kills joint cells and then examined the effects on macrophage activation responses following engulfment of dead and dying cells. Stimulation of anti-inflammatory cytokine production (TGF-β) and suppression of pro-inflammatory cytokine secretion (TNF-α, IL1-β, and IL-6) were observed in this model compared to cells that died by necrotic pathways, consistent with previous reports (38, 41). Efferocytosis has been described as an important step to resolving inflammation and restoring normal tissue function, with macrophages playing an integral role in the maintenance of joint homeostasis (38, 41). The differential cytotoxicity of amikacin to cell types was also demonstrated, with leukocytes overall, and particularly neutrophils, being sensitive to amikacin-induced cell death at concentrations that would be easily achieved in synovial fluid with the doses commonly used in equine practice. The susceptibility to antibiotic killing of leukocytes likely accounts for the lack of differences observed in the clinicopathological parameters of synovial fluid and could mask the inflammatory effects of antibiotic-induced cytotoxicity. These findings indicate that particular attention should be paid to the off-target cytotoxic effects of certain antibiotics when used for local treatment of infections due to the potential for inducing tissue injury and immune suppression.

Limitations to the study design include the small horse sample size, evaluation in normal vs. inflamed joints, lack of synovial fluid collection at later time points for biomarker analysis to investigate when inflammatory cytokine concentrations returned to baseline, and the lack of histopathologic evaluation of synovial tissues. Obtaining a bigger sample size and performing additional sampling of the synovial fluid past 24 h were not possible due to financial constraints, but may have revealed continued increases in the biomarkers of collagen degradation and potentially statistically significant elevations in the CRP values, as has been previously reported (34). Furthermore, synovial fluid sampling at later time points and evaluation of the histopathology of synovial tissues may have provided further information as to whether intra-articular amikacin administration resulted in long-term joint damage or was only associated with a transient increase in pro-inflammatory cytokines and cartilage degradation products. The timing and frequency of synovial fluid sampling were performed to emphasize pharmacokinetic analysis of amikacin concentrations in the synovial fluid, where a greater number of early time points allowed determining synovial fluid concentration inflection points and the single 24-h time point allowed calculating β and the terminal half-life slope, as has been previously described (13, 34). Synovial fluid sampling may have transiently affected the other parameters evaluated (e.g., clinicopathological parameters), as has been previously reported (42), but the study design, which included a control sample, should have mitigated this potential complication. Comparison of the elevations in inflammatory biomarkers and cartilage degradation products to long-term histopathological findings may have provided some clarity as to whether the detrimental effects associated with amikacin injection reported here are temporary or enduring, as cytological evaluation of the synovial fluid has been previously reported to be more sensitive than histology (43). Further *in vivo* evaluation of antibiotic distribution and cytotoxicity when co-administered with other medications intra-articularly or in inflamed or infected joints is warranted. In investigating the use of amikacin in the treatment of infected joints, evaluation of additional parameters not reported here, such as synovial fluid pH and glucose and lactate levels, may be important in monitoring response to therapy (44, 45). This work will serve as a platform from which further investigations of intra-articular antibiotic use will build.

The horse represents a valuable large animal preclinical model for human joint disease, including treatment of septic arthritis (46–53). The joint volume and cartilage thickness of horses more closely approximate that of human cartilage compared to other animal models, and the larger volume of synovial fluid within equine joints allows for a greater ease of synovial fluid sampling for the analysis of increased outcome parameters (46–50). Equine articular cartilage is subject to loading forces of similar or greater magnitude than human cartilage, which may have important implications when evaluating intra-articular therapies (46–48). The formation of bacterial biofilm aggregates in equine synovial fluid as a substitute for human synovial fluid was recently described *in vitro*, and clinical evidence of septic arthritis as a disease process in horses is well-documented, providing further evidence to support the use of the equine preclinical model as both a translational and clinically relevant model for human joint disease (52, 54).

This study demonstrated that the concentrations of amikacin administered intra-articularly in horses reached and sustained the therapeutic levels for most common equine pathogens for all doses assessed while eliciting an increased production of cartilage degradation products and pro-inflammatory biomarkers. The effects of amikacin on equine cartilage *in situ* and in the presence of inflammation and sepsis warrant further investigation, but these findings indicate that amikacin can penetrate the cartilage to elicit a dose-dependent cytotoxicity *in vivo*. In conclusion, the use of intra-articular antibiotics has the potential to augment the current treatment regimens for septic arthritis, but induces joint inflammation and cartilage degradation at higher doses *in vivo*. Decisions on whether to inject cytotoxic antibiotics such as aminoglycosides intra-articularly and what doses to use should take into account the potential harm that antibiotics may cause and consider lower doses than those previously reported in equine practice.

## Data Availability Statement

The raw data supporting the conclusions of this article will be made available by the authors, without undue reservation.

## Ethics Statement

The animal study was reviewed and approved by Institutional Animal Care and Use Committee of Colorado State University (protocol #19-9058A).

## Author Contributions

LP, LG, SD, LC, DH, and JS conceptualized and designed the study. LP, LG, SD, LC, JP, GP, PL, GG, DG, and AR acquired the data. LP, LG, SD, LC, GG, PL, JP, DG, AR, and JS analyzed and interpreted the data. LP, LC, and GG drafted the manuscript. All authors contributed to and approved the submitted version of the manuscript.

## Conflict of Interest

The authors declare that the research was conducted in the absence of any commercial or financial relationships that could be construed as a potential conflict of interest.
